# Immunogenicity of idursulfase and clinical outcomes in very young patients (16 months to 7.5 years) with mucopolysaccharidosis II (Hunter syndrome)

**DOI:** 10.1186/s13023-015-0265-2

**Published:** 2015-04-24

**Authors:** Arian Pano, Ann J Barbier, Bonnie Bielefeld, David AH Whiteman, David A Amato

**Affiliations:** Shire, 300 Shire Way, Lexington, MA 02421 USA; Current address: Vertex Pharmaceuticals, Cambridge, MA USA

**Keywords:** Mucopolysaccharidosis II, Idursulfase, Antibodies, Treatment outcome

## Abstract

**Background:**

Twenty-eight treatment-naïve mucopolysaccharidosis II patients (16 months–7.5 years) received 0.5 mg/kg idursulfase weekly for one year in NCT00607386. Serum anti-idursulfase immunoglobulin G antibodies (Abs) were seen in 68% of patients.

**Methods:**

This post hoc analysis examined the relationship between Ab status, genotype, adverse events (AEs), and efficacy. Event rate analyses, time-varying proportional hazards (Cox) modeling, and landmark analyses were performed to evaluate the relationship between Ab status and safety. We calculated the cumulative probability of AEs by genotype to evaluate the relationship between genotype and safety. Urinary glycosaminoglycan (uGAG) concentration, index of liver size, and spleen volume were compared by Ab status and genotype.

**Safety results:**

The overall infusion-related AE (IRAE) rate was higher in Ab+ patients than in Ab− ones. However, the rate was highest *before* Abs developed, then decreased over time, suggesting that Abs did not confer the risk. A landmark analysis of patients who were IRAE-naïve at the landmark point found that Ab+ patients were no more likely to experience post-landmark IRAEs than were Ab− patients. In the genotype analysis, all patients in the complete deletion/large rearrangement (CD/LR) and frame shift/splice site mutation (FS/SSM) groups seroconverted, compared with only one-third of patients in the missense mutation (MS) group (*p* < 0.001). The cumulative probability of having ≥1 IRAE was 87.5% in the CD/LR group and 46.2% in the MS group, with a shorter time to first IRAE in the CD/LR group (*p* = 0.004).

**Efficacy results:**

Ab+ patients had a reduced response to idursulfase for liver size and uGAG concentration, but not for spleen size. However, when percent change from baseline in liver size and in uGAG level at Week 53 were adjusted for genotype, the difference was significant only for neutralizing Ab+ groups. In the genotype analysis, the CD/LR and FS/SSM groups had a reduced response in liver size and uGAG concentration compared with the MS group.

**Conclusions:**

Safety outcomes and spleen size response on idursulfase treatment appeared to be associated with genotype, not Ab status. Liver size and uGAG response on idursulfase treatment at Week 53 appeared to be associated with both neutralizing Ab status and genotype.

## Background

Mucopolysaccharidosis II (MPS II, Hunter syndrome; OMIM 309900) is a rare, X-linked lysosomal storage disorder caused by a deficiency in the enzyme iduronate-2-sulfatase (I2S), leading to the accumulation of glycosaminoglycans (GAGs) within lysosomes [1,2]. Patients generally appear normal at birth, but develop characteristic, multi-systemic signs and symptoms beginning during early childhood [2,3]. Disease manifestations progress over time, leading to significant morbidity and early mortality [4,5]. In addition, about two-thirds of patients experience progressive cognitive impairment [6]; such patients are said to have the severe phenotype.

Enzyme replacement therapy consists of administration of the missing enzyme in recombinant form, and this has been successful in a number of lysosomal storage diseases, such as Fabry, Gaucher, MPS I, MPS II, MPS IV, and MPS VI [7]. Inevitably, such treatment will lead to a certain level of immunogenicity in patients who produce no endogenous enzyme. This has been most dramatically shown in Pompe disease, where patients with no cross-reactive immune material (CRIM) have a high rate of antibody (Ab) production to the exogenously administered enzyme [8,9], and in MPS IV, where Ab production was universally observed in the patients in the pivotal trial [10]. The real question; however, is not so much the rate of seroconversion, but the clinical impact of these Abs, especially of neutralizing antibodies (NAbs), which would be expected to mitigate the clinical efficacy of the treatment. In Pompe disease especially, there is clear evidence that the humoral Ab response in CRIM-negative patients is associated with worse outcomes [9], whereas in MPS II and MPS I, the focus has been on the association between biomarkers and Abs [11,12].

In MPS II, two clinical trials have been performed with treatment-naïve patients. The first of these studies, TKT-ELA-024 (NCT00069641), was the topic of a previously published analysis [11]. This analysis, which comprised data from 63 patients who received enzyme replacement therapy with intravenous idursulfase 0.5 mg/kg weekly in the phase II/III study and/or the extension study examined the relationship between Ab status and safety and efficacy outcomes in an attenuated, treatment-naïve population of patients 5 years of age and older [11]. We found that Ab positivity did not appear to lessen the clinical response to idursulfase as assessed by the 6-minute walk test, and patients who were Ab positive (Ab+) did not appear to have an increased risk of serious adverse events (SAEs). Infusion-related adverse events (IRAEs) were about two-fold more likely in patients who became Ab+ on treatment; however, most of the risk occurred before Abs developed and could be ameliorated after a first reaction through subsequent use of previously published preventative measures [13]. A genotype analysis in the 36 patients with available data found that patients with nonsense or frameshift mutations appeared more likely to develop Abs, to experience IRAEs, and to have a slightly reduced urinary GAG (uGAG) response than those with missense mutations (MS). These findings suggest that Abs are not a driver of clinical outcomes, but instead may be a marker for genotype.

One inherent limitation to the post hoc analysis described above was that only patients over the age of 5 years and only those without cognitive impairment were included in the original phase II/III trial and extension study. None of the patients with available genotype data in these trials had a complete deletion/large rearrangement (CD/LR); the genotype group most frequently associated with cognitive impairment, the hallmark of the severe form of MPS II. The current paper expands the analysis of the association between Abs, genotype, and clinical outcomes by describing the results of a similar analysis of an open-label clinical study (HGT-ELA-038; NCT00607386) in which the primary objective was to determine the safety of once-weekly intravenous dosing of idursulfase 0.5 mg/kg in patients with MPS II up to the age of 5 years with phenotypes ranging from attenuated to severe [14]. Secondary endpoints included uGAG levels, growth, liver size, and spleen size. The safety profile of idursulfase in this study was similar to that seen in previous clinical trials [15,16], and treatment was associated with decreases in uGAG levels, liver size, and spleen volume. A total of 19/28 patients (67.9%) tested positive for anti-idursulfase immunoglobulin G (IgG) Abs at least one time point during the study. In this report, we extend our investigation into the association between anti-idursulfase Ab status, genotype, and the efficacy and safety of idursulfase via a post hoc analysis of data from the 28 patients who were enrolled in HGT-ELA-038.

## Methods

### Patients and study design

HGT-ELA-038 enrolled male patients ≤5 years of age at screening with a clinically and biochemically confirmed diagnosis of MPS II [14]. Exemptions to enter the study were granted to 4 patients aged >5 years (6.5 years, 7.5 years, 6.3 years, and 6.2 years). These patients met all other enrollment criteria. They had been identified at ≤5 years of age, but their study-site initiation was delayed due to logistical issues in study start-up. Exclusion criteria have been previously reported. Patients received idursulfase 0.5 mg/kg by intravenous infusion every week (±3 days) for 52 weeks. The population for the current post hoc analysis was the safety population of HGT-ELA-038, which included all enrolled patients who received at least one dose or any portion of a dose of idursulfase during the study.

HGT-ELA-038 was conducted in compliance with international guidelines and appropriate local country regulations. Written informed consent was given by the parents/guardians. The protocol and informed consent documents were approved by the institutional review board and/or independent ethics committee at each study site. The work described in this manuscript represents new statistical analyses of immunogenicity (antibody) test results that were generated during HGT-ELA-038. No new blood samples were obtained in order to perform the current analyses, and no existing blood samples were re-analyzed for Ab titer.

### Ab measurements

As part of the HGT-ELA-038 study design, blood samples were screened by Shire Bioanalytics, Lexington, MA, USA, for the detection of serum idursulfase IgG Abs using the conformation-specific antibody (CSA) or enzyme-linked immunosorbent (ELISA) assays [16]. All samples meeting the CSA or ELISA cut points were confirmed by a radioimmunoprecipitation (RIP) assay. All Ab+ samples were analyzed for NAbs with both an *in vitro* activity-neutralizing assay [17] or a cell-based internalization assay [18].

### Ab status

The following definitions were used for Ab status:

- Ab+: At least one serum specimen had measurable anti-idursulfase IgG Ab by either CSA or ELISA, confirmed by RIP, regardless of Ab status at any subsequent visits. A patient was considered to be Ab+ at a given week if, by this time point, the patient had at least one visit at which there were measurable Abs.
- Persistently Ab+ (PAb+): There were 3 or more consecutive visits at which the patient was Ab+, regardless of the Ab status at any subsequent visits. A patient was considered to be PAb+ at a given week if, by this time point, the patient had the first of 3 or more consecutive visits at which there were measurable IgG anti-idursulfase Abs.
- Neutralizing Ab+ (NAb+): At least one serum specimen was positive at any time during the study for NAbs on either the activity-NAb assay or a cell-based internalization Ab assay, regardless of the Ab status at any subsequent visits. A patient was considered to be NAb+ at a given week if, by this time point, the patient had at least one visit at which there were measurable NAbs measured by either assay.
- Persistently NAb+ (PNAb+): There were 3 or more consecutive visits at which the patient was NAb+, regardless of NAb status at subsequent visits. A patient was considered to be PNAb+ at a given week if, by this time point, the patient had the first of 3 or more consecutive visits at which there were measurable NAbs by either the activity-NAb assay or a cell-based internalization Ab assay.
- Antibody Negative (Ab−): A patient was considered Ab− if all IgG anti-idursulfase Ab tests were negative for the patient throughout the treatment period. A patient was considered to be Ab− at a given week if, by this time point, there were no previous visits at which the patient had measurable IgG Abs, even if the patient later became Ab+.
- Not PAb+: Patients did not meet the criteria to be PAb+ at any time. Note that these patients could be Ab−, Ab+, or NAb+.
- NAb Negative (NAb−): A patient had no positive serum specimen during the study by either an activity-NAb assay or a cell-based internalization Ab assay. Note that these patients could be Ab−, Ab+, or PAb+.
- Not PNAb+: Patients did not meet the criteria to be PNAb+ at any time. Note that these patients could be Ab−, Ab+ or NAb+.

### Time point assignment for Ab status changes

Testing for anti-idursulfase Abs in HGT-ELA-038 was performed using serum samples collected at baseline and at scheduled visits for Study Weeks 9, 18, 27, 36, 45, and 53. Note that Study Week visits in which idursulfase infusions were administered were scheduled to occur at 7-day intervals; however, variations of ±3 days were permitted for actual visit dates. Therefore, a Study Week visit of a certain number (e.g., Week 27) for a given patient may not have occurred at precisely the corresponding number of calendar weeks after treatment start. The protocol specified additional (unscheduled) sample collections if there was a suspicion of an IRAE. These unscheduled collections were assigned a time point in Calculated Weeks by taking the date of the sample collection minus the date of the baseline visit and dividing by 7.

Because Ab testing was performed at discrete time points, the exact day on which a patient became Ab+ could not be determined. We used 2 guiding principles to develop an algorithm to assign seroconversion dates. First, all patients who became seropositive in our study had done so by the first scheduled Ab sampling date at the Study Week 9 visit. This meant that all patients became seropositive at an unknown time point sometime before the Study Week 9 visit. Second, it takes several weeks to mount a measurable IgG response to a newly introduced antigen [19].

Therefore, we developed an algorithm as follows:

- If there were no unscheduled, positive samples taken before the Study Week 9 visit, then the date of seroconversion was imputed to be Calculated Week 4.5; that is, halfway between the date of the baseline visit and the date of the Study Week 9 visit (i.e., the first documented seropositive sample).
- If a patient had an unscheduled, positive sample collection prior to Calculated Week 4.5, then the patient was considered to have become positive on the date of the sample collection. For example, if the patient had an unscheduled, positive serum sample at Calculated Week 3, then he was considered to have seroconverted on that date.
- If the patient had an unscheduled, positive sample collection after Calculated Week 4.5, but prior to Study Week 9, then the patient was considered to have become seropositive at the midpoint between the last negative and first positive sample. If the midpoint was before Calculated Week 4.5, however, then Week 4.5 was assigned as the seroconversion date. For instance, if a patient had an unscheduled, positive sample at Study Week 5, then the patient was considered to have become seropositive at Calculated Week 4.5 (not at Week 2.5).

### Safety assessments

In HGT-ELA-038, treatment-emergent adverse events (TEAEs) were defined as adverse events (AEs) that occurred on or after the first dose of idursulfase until 30 days after the completion of treatment. IRAEs were defined as TEAEs that began on the day of the infusion or the next day and were judged as possibly or probably related to the study drug. SAEs were defined as any TEAEs that were life-threatening or resulted in death, inpatient hospitalization, prolongation of existing hospitalization, a persistent or significant disability/incapacity, or a congenital anomaly/birth defect. Important medical events that may not result in death, be life-threatening, or require hospitalization were considered to be SAEs when, based upon appropriate medical judgment, they may have jeopardized the patient and may have required medical or surgical intervention to prevent one of the outcomes listed above.

### Efficacy assessments

Efficacy variables in HGT-ELA-038 included uGAG concentration, index of liver size (ILS), and spleen volume as previously reported [14]. Urine samples for determination of uGAG levels and urine creatinine were collected at baseline and at Study Weeks 18, 36, and 53. Urinary GAG levels were normalized to urine creatinine and were reported as μg GAG/mg creatinine. Abdominal ultrasound examinations were conducted at local centers at baseline and at Study Weeks 18, 36, and 53 to assess the ILS [20]. With the patient in the supine position, the section level of the liver in the anterior axillary line (AAL [cm]), medioclavicular line (MCL [cm]), and the perpendicular section plane in the sternal line (STL [cm]) were measured 3 times. The ILS was calculated using the following formula [20]: ILS (cm^2^) = 0.2618 * (AAL^2^ + MCL^2^ + STL^2^). Spleen volume was assessed by abdominal ultrasound examinations conducted at baseline and at Study Weeks 18, 36, and 53. With the patient in the right-recumbent position, the length (L), depth of length (DL), breadth (B), and depth of breadth (DB) were also measured 3 times (unit of measure: cm). The spleen volumes were calculated using the following formula for an ellipsoid [20]:

$$ \mathrm{Spleen}\ \mathrm{volume}\ \left(\mathrm{c}{\mathrm{m}}^3\right)=0.523\ *\ \mathrm{L}\ *\ \mathrm{B}\ *\ \left(\mathrm{D}\mathrm{L} + \mathrm{D}\mathrm{B}\right)/2. $$

### Data analysis: safety and efficacy outcomes by Ab status

Landmark analyses were performed to evaluate the relationship between anti-idursulfase IgG Abs and safety outcomes. A landmark analysis divides patients into 2 or more groups (e.g., PAb+ vs Not PAb+) at a fixed point in time (the landmark point). These groups are then compared prospectively on the basis of outcomes (e.g., IRAEs, SAEs) that occur subsequent to the landmark point [21]. The median time to the first event after the landmark point of Calculated Week 4.5 was estimated using the Kaplan-Meier method and compared between Ab status subgroups using the log-rank test. No analyses were performed on Ab subgroups having fewer than 3 seropositive patients (~10% of the study population) for that specific Ab status.

In some cases, a time-varying proportional hazards regression (Cox) model was also used to assess the association between Ab status and time to the first occurrence of a specific AE. Patients who had no event were censored as of the time of their last visit. A relative risk greater than 1 indicates that patients who have a positive Ab status are at greater risk of the event than patients who do not.

Event rate analyses were conducted to explore the relationship between Ab status and AEs. Event rate analyses were stratified by Ab status. For a patient who was always Ab−, the event rate was the number of events observed divided by the total time on study (defined as U). For a patient who became Ab+ during the study, T was defined as the date of seroconversion as calculated following the algorithms described above. Using this definition for T, the negative period event rate is the total number of events occurring prior to time T divided by the total time on study while Ab− (time = T – 1 day). The positive period event rate is the total number of events that occurred at or after time T divided by the time on study while Ab+ (U – T + 1 day).

To assess the relationship between Ab status and efficacy outcomes, we compared ILS actual values, MCL z-scores, spleen volumes, and normalized values for uGAG at Study Weeks 18, 36, and 53 by Ab status at the landmark point as described above for landmark analyses using the Wilcoxon rank sum test. Because genotype may be related to both Ab status and efficacy outcomes, we also conducted analyses of the percent change from baseline in ILS, spleen volume, and uGAG concentration at Week 53 by Ab status at the landmark point while adjusting for genotype using analysis of covariance. This was done to determine whether there was any residual relationship between outcomes and Ab status beyond that explainable by genotype.

### Data analysis: safety and efficacy outcomes by genotype

Patients with available data were classified into the MS group, the CD/LR group, or the frameshift/splice site mutation (FS/SSM) group, defined as follows:

- MS: A mutation that results in nucleotide changes in the *IDS* gene that introduces a single amino acid residue change in the I2S enzyme.
- CD/LR: A mutation that results in complete deletions or large rearrangements to the *IDS* gene.
- FS: A mutation that introduces a frame shift in the *IDS* gene due to nucleotide deletions or insertions.
- SSM: A mutation that inserts or deletes a number of nucleotides in the *IDS* gene in a specific site at which splicing of an intron takes place during the processing of precursor messenger RNA into mature messenger RNA.

Note that one patient with the nonsense mutation R443X, which leads to a stop codon in the ninth and final exon of the *IDS* gene, was grouped into the MS group.

Safety outcomes were analyzed by genotype group to explore the relationship between genotype and AEs. The time to a first TEAE, SAE, or IRAE was calculated using the Kaplan-Meier method [22].

For efficacy outcomes, the actual values for the ILS, spleen volumes, and the actual normalized value for uGAG, were summarized for each visit (baseline and Study Weeks 18, 36, and 53) by genotype. Comparisons between genotypes were assessed at each visit using the Wilcoxon rank sum test for the observed value and an analysis of covariance (ANCOVA) model with baseline value included in the model as the covariate for change and percent change from baseline.

## Results

### Study population and genotype

The details of the original study have been previously published [14]. In brief, 28 patients aged 16 months to 7.5 years (mean 4.0 years) were enrolled; one patient (3.6%) discontinued after 4 weeks of treatment due to compliance issues, but was followed for 16 weeks. The mean age at diagnosis was 3.5 years (0.2–6.5 years). There were no clear differences in age at diagnosis or at enrollment between the patients who became Ab+ and those who remained negative throughout the study.

Genotype data were available for 27 patients. Patients were categorized into one of three groups based on genotype (see Ab Status in Methods). These groups were chosen based on the anticipated impact on endogenous protein levels and functionality. The first group includes patients with a CD/LR mutation. These patients lack the *IDS* gene and produce no endogenous enzyme. It is likely that an initial exposure to idursulfase could result in a robust immune response since the enzyme would be viewed as foreign by the body’s immune system. The second group contains patients with FS/SSM. Such mutations could be expected to lead to protein products that range from those that are very short and non-functional to those that are somewhat longer and may retain some amount of residual enzyme activity. The third group includes patients with MS, which would be expected to produce some endogenous protein products with varying degrees of integrity and functionality. We anticipated that since the patients in the MS group have had some exposure to I2S enzyme, albeit in very low quantities and in mutated form, there would be a lower incidence of an Ab response to idursulfase than would be seen in the other genotype groups. Among the 27 patients with genotype data, 8 were in the CD/LR group; 6 were in the FS/SSM group; and 13 were in the MS group.

### Ab status

**Figure 1:**
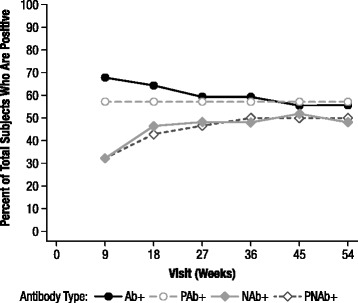
Ab+, PAb+, NAb+, and PNAb+ percentages of total subjects by Study Week visit in the primary analysis population. Percent of total subjects that are Ab+, PAb+, NAb+, and PNAb+ by Study Week visit in the primary analysis population. For the Ab+, and NAb+ patients, the percentage of patients who have Abs or NAbs, respectively, at that particular study visit are given. For PAb+ and PNAb+ patients, the cumulative percentage of patients is shown. A patient was considered to be PAb+ or PNAb+ at a given week if, by this time point, the patient had the first of 3 or more consecutive visits at which there were measurable anti-idursulfase Abs/NAbs. *Ab+*, antibody positive; *NAb+*, neutralizing antibody positive; *PAb+*, persistently antibody positive; *PNAb+*, persistently neutralizing antibody positive.

An overall summary of Ab status is presented in Table 1. When we analyzed Ab status by Study Week visit, we found that all Ab+ and PAb+ patients had seroconverted by Study Week 9, since PAb+ status was considered to occur at the time of the first of 3 consecutive positive serum samples. All patients who became NAb+, did so by Study Week 27; and all those who became PNAb+, did so by Study Week 36 (Figure 1). Of the 15 patients who became NAb+ (Table 1), Abs from 14 displayed neutralizing activity and Abs from all 15 displayed cellular uptake inhibiting activity. The calculated time of seroconversion was the landmark time used in all analyses.

**Table 1:** **Overall Ab status and relationship between IgG and NAbs in the primary analysis population**

**N (%)**	**Ab+ (n = 19)**	**PAb+ (n = 16)**	**NAb+ (n = 15)**	**PNAb+ (n = 14)**	**Total (n = 28)**
Ab+	--	16 (100)	15 (100)	14 (100)	19 (68)
PAb+	16 (84)	--	14 (93)	14 (100)	16 (57)
NAb+	15 (79)	14 (88)	--	14 (100)	15 (54)
PNAb+	14 (74)	14 (88)	14 (93)	--	14 (50)

*Ab+,* antibody positive; *IgG*, immunoglobulin G; *NAb+,* neutralizing antibody positive; *PAb+,* persistently antibody positive; *PNAb+,* persistently neutralizing antibody positive.

### Safety assessments and relationship with Abs status

#### IRAEs

**Figure 2:**
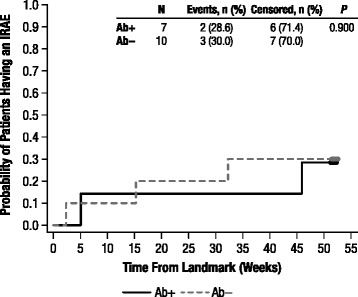
Kaplan-Meier plot of time to the first IRAE after the landmark point for patients with no IRAEs prior to the landmark point, by PAb status at the Study Week 9 visit. *IRAE*, infusion-related adverse event; *PAb+*, persistently antibody positive; *PAb−*, persistently antibody negative.

We performed a landmark analysis to determine whether Ab status was associated with a shorter time to first IRAE after the landmark point (see Data Analysis in Methods). Only patients who were IRAE-naïve at the landmark point were included because the risk of IRAEs changes over time, given that standard preventive measures are implemented after a first IRAE. We found that PAb+ patients were no more likely to experience future IRAEs than were Not PAb+ patients (*p* = 0.90, Figure 2). Similar results were seen for Ab+ vs Ab − patients (*p* = 0.46); NAb+ vs NAb− patients (*p* = 0.52); and PNAb+ vs Not PNAb+ patients (*p* = 0.52).

Next, an IRAE event rate analysis revealed several interesting patterns. First, IRAE event rates were lowest among patients who were always Ab− during the study (0.014 events/week, 95% CI: 0.004–0.024 events/week). Second, as expected, the IRAE event rate fell over time for all Ab status groups, likely due to preventive measures implemented after a first IRAE as described above. The third pattern we observed was that, among patients who developed Abs, the event rates were highest *before* those Abs developed. For example, the rate was 0.239 events/week (95% CI: 0.125–0.352 events/week) for eventual Ab+ patients during the period before becoming Ab+ and 0.036 events/week (95% CI: 0.024 – 0.048) after becoming Ab+. Similar results were seen for PAb+, NAb+, and PNAb+ patients (data not shown). These findings suggest that rather than Abs being the cause of IRAEs, certain patients may intrinsically possess a characteristic that predisposes them to both Ab formation and IRAEs.

#### TEAEs and SAEs

The majority of TEAEs and SAEs in this study were considered to be related to manifestations commonly seen in patients with MPS II (e.g., respiratory infections). All patients experienced TEAEs, and the event rate did not significantly differ according to Ab status (data not shown). We conducted landmark analyses to determine whether the time to a first TEAE after the landmark point differed by Ab status at the landmark point, but we found no significant differences (data not shown). Similarly, the time-varying proportional hazards (Cox) model found that Ab status was not associated with any statistically significant overall increase in TEAE risk (data not shown).

The SAE rates were low overall. A total of 13/28 patients (46.4%) experienced a total of 38 SAEs (Table 2) in study HGT-ELA-038. Of these, 4 patients (14.3%) reported 10 SAEs that were considered possibly or probably related to the study drug; all of these SAEs were also IRAEs. All of the events were considered by the investigators to be mild or moderate in severity. The proportion of patients with at least one SAE was always slightly, but not statistically significantly, higher in the groups with an Ab+ status (Ab+, PAb+, Nab+) than in the corresponding Ab− groups. Landmark analyses indicated that the time to the first SAE after the estimated time point of seroconversion was numerically shorter for patients with a positive Ab status, but again the differences were not statistically significant. Note that in the landmark analyses, only those first SAEs which occurred after the landmark (28 SAEs out of 38 total) were considered. The time-varying proportional hazards (Cox) model found that, although patients with a positive Ab status had a numerically higher risk of SAEs than their Ab− counterparts, these differences were not statistically significant (data not shown).

**Table 2:** **SAEs by patient**

**Patient information**			**SAE information**			
**ID**	**Genotype group**	**Ab status overall**	**Preferred term**	**Relationship to study drug**	**IRAE**	**Included in landmark analyses?** ^**a**^
1	FS/SSM	PNAb+	Bronchopneumonia	Not related	No	Yes
			Bronchospasm	Not related	No	Yes
2	CD/LR	PAb+	Respiratory distress	Probably related	Yes	No^b^
			Pulmonary hypertension	Not related	No	Yes
3	MS	Ab−	Bronchopneumonia	Not related	No	Yes
			Hypoxia	Not related	No	Yes
			Respiratory distress	Not related	No	Yes
4	FS/SSM	PNAb+	Microcytic anemia	Not related	No	Yes
			Pneumonia	Not related	No	Yes
5	CD/LR	PNAb+	Rash	Probably related	Yes	No^b^
			Pyrexia	Probably related	Yes	No^b^
			Pyrexia	Probably related	Yes	Yes
			Pyrexia	Probably related	Yes	No^c^
			Pyrexia	Probably related	Yes	No^c^
			Irritability^d^	Not related	No	Yes
6	MS	Ab−	Skin hypertrophy	Not related	No	Yes
7	CD/LR	PNAb+	Urticaria	Possibly related	Yes	No^b^
			Pyrexia	Possibly related	Yes	No^b^
			Urticaria	Possibly related	Yes	No^b^
			Respiratory tract infection	Not related	No	Yes
			Respiratory tract infection	Not related	No	No^c^
			Ear infection	Not related	No	Yes
			Viral pharyngitis	Not related	No	Yes
			Pneumonia	Not related	No	Yes
			Urticaria	Not related	No	Yes
			Atonic seizures	Not related	No	Yes
			Edema peripheral	Not related	No	Yes
8	FS/SSM	Ab+	Gastrointestinal infection^d^	Not related	No	Yes
9	Unknown	PNAb+	Food poisoning^d^	Not related	No	Yes
			Upper respiratory tract infection	Not related	No	Yes
			Muscle contracture	Not related	No	Yes
10	CD/LR	PNAb+	Pyrexia^d^	Probably related	Yes	Yes
11	CD/LR	PNAb+	Asthma	Not related	No	Yes
			Bronchopneumonia	Not related	No	Yes
			Otitis media	Not related	No	Yes
12	CD/LR	PNAb+	Hematoma	Not related	No	Yes
13	FS/SSM	PNAb+	Catheter site hematoma	Not related	No	No^b^
			Otitis media	Not related	No	Yes

*Ab+*, antibody positive; *Ab−*, antibody negative; *CD/LR*, complete deletion/large rearrangement; *FS/SSM*, frameshift/splice site mutation; *IRAE*, infusion-related adverse event; *landmark*, estimated time point of change in Ab status; *MS*, missense mutation; *PAb+*, persistently antibody positive; *PNAb+*, persistently neutralizing antibody positive, SAEs, serious adverse events.

^a^Only 28 of the 38 total SAEs that occurred during HGT-ELA-038 were included in the landmark analyses investigating the relationship between Ab status, genotype, and risk of SAEs. SAEs were excluded from the landmark analyses if they occurred before the landmark point (the estimated time point of change in Ab status; see Methods) or if they were not the first occurrence of an SAE for a given patient.

^b^Occurred before the landmark point.

^c^Not the first occurrence for this patient.

^d^Patients were hospitalized for social reasons (i.e., convenience) rather than for medical reasons.

#### Relationship between genotype group, Ab status, and risk of IRAEs and SAEs

We next examined the association between genotype, the development of Abs, and the risk of IRAEs or SAEs. We found that patients in the CD/LR group were the most likely to develop Abs, while patients in the MS group were the least likely to develop Abs (Table 3). Indeed, patients who remained Ab− throughout the study were only found in the MS group. These Ab status differences were statistically significant for the CD/LR group vs the MS group (*p* ≤ 0.005 for each of Ab+, PAb+, NAb+, and PNAb+), as well as for the MS group vs the FS/SSM group (*p* < 0.05 for all, except PAb+). The differences between the CD/LR and FS/SSM groups in regards to Ab status were not statistically significant.

**Table 3:** **Ab status at end of study by genotype class (n = 27)** ^**a**^

	**Mutation group, n (%)**			**Statistical comparison**		
**Ab status**	**CD/LR (n = 8)**	**FS/SSM (n = 6)**	**MS (n = 13)**	**CD/LR vs FS/SSM** ^**b**^	**CD/LR vs MS** ^**b**^	**FS/SSM vs MS** ^**b**^
Ab+	8 (100)	6 (100)	4 (31)	*p* = 1.000	*p* = 0.005	*p* = 0.011
PAb+	8 (100)	4 (67)	3 (23)	*p* = 0.165	*p =* 0.001	*p* = 0.129
NAb+	7 (88)	5 (83)	2 (15)	*p* = 1.000	*p =* 0.002	*p* = 0.010
PNAb+	7 (87.5)	4 (66.7)	2 (15.4)	*p* = 0.538	*p =* 0.002	*p* = 0.046

*Ab+*, antibody positive; *CD/LR*, complete deletion/large rearrangement; *FS/SSM*, frameshift/splice site mutation; *MS*, missense mutation; *NAb+*, neutralizing antibody positive, *PAb+*; persistently antibody positive; *PNAb+*, persistently neutralizing antibody positive.

^a^Genotype data not available for one patient enrolled in HGT-ELA-038.

^b^ *p*-value from Fisher’s exact test.

We calculated the overall cumulative probability of patients having at least one IRAE by genotype group and found that, by end of study, it was highest for patients in the CD/LR group (87.5%) and lower for patients in the FS/SSM group (50.0%) or the MS group (46.2%). When we compared the time to development of a first IRAE among genotype groups, we found that the time was significantly shorter for the CD/LR group than it was for the MS group (*p* = 0.004). However, we found no statistically significant differences in the time to development of a first IRAE when we compared the FS/SSM group with either the CD/LR (*p* = 0.11) or the MS group (*p* = 0.86).

For SAEs, the cumulative probability of having at least one SAE by genotype group was highest for the CD/LR and the FS/SSM groups (75.0% each) and lowest for the MS group (15.4%). When we compared the time to development of a first SAE among groups, we found that the time was significantly shorter for the CD/LR (*p* = 0.003) and FS/SSM groups (*p* = 0.007) than it was for the MS group.

These findings indicate that the CD/LR genotype is associated with the highest risk of IRAEs and SAEs. Because all patients with this genotype invariably developed Abs (Table 3), any apparent IRAE and SAE risk differences observed in the analysis by Ab status may be confounded by this association. Additionally, many SAEs were related to MPS II disease progression. It is plausible that patients with the CD/LR genotype simply had a higher disease burden, which manifested as SAE-qualifying events. This is supported by the fact that when the time-varying proportional hazards (Cox) model for risk of a first SAE by Ab status (see TEAEs and SAEs, above) was adjusted for genotype, the numerically greater (but not statistically significant) risk associated with positive Ab status was no longer apparent.

### Efficacy assessments by Ab status and genotype group

#### Liver size

**Figure 3:**
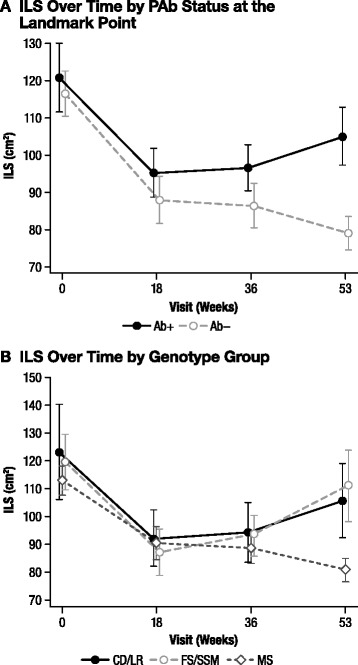
The ILS over time by PAb status and genotype group. **A)** ILS over time by PAb status at the landmark point. Week 53, PAb+ vs PAb−, *p* = 0.025. **B)** ILS over time by genotype group. Week 53, FS/SSM vs MS, *p* = 0.049. *CD/LR*, complete deletion/large rearrangement; *FS/SSM*, frameshift/splice site mutation; *ILS*, index of liver size; *MS*, missense mutation; *PAb+*, persistently antibody positive; *PAb−*, persistently antibody negative.

To examine the relationship between Ab status and liver size during idursulfase treatment, we compared the mean ILS score over time by Ab status at the landmark point. All patient groups experienced an initial decrease in mean ILS score after treatment start (Figure 3A). PAb+ patients had a larger mean observed ILS score at Week 53 than did Not PAb+ patients (*p* = 0.025). Similar results were seen for Ab+ vs Ab− (*p* = 0.008), NAb+ vs NAb− (*p* = 0.001), and PNAb+ vs Not PNAb+ (*p* = 0.001) patients (data not shown).

Because genotype may be related to both Ab status and efficacy outcomes, percent change from baseline in ILS at Week 53 was also compared between Ab status groups, adjusted for genotype using analysis of covariance. This was done to determine whether there was any residual relationship between liver size outcome and Ab status beyond that explainable by genotype. After adjustment for genotype, only the comparisons involving NAb status groups remained significant at Week 53 (Ab+ vs Ab− adjusted *p* = 0.365; PAb+ vs. Not PAb+ adjusted *p* = 0.193; NAb+ vs NAb− adjusted *p* < 0.001; PNAb+ vs Not PNAb+ adjusted *p* < 0.001; data not shown).

To assess the relationship between genotype and liver size during treatment with idursulfase, we plotted the mean observed ILS score over time by genotype and found that it decreased over time in all genotypes, with the most pronounced decrease observed in the MS group (Figure 3B). At Week 53, the mean observed ILS score was significantly lower for the FS/SSM group than it was for the MS group (*p* = 0.049). Other comparisons between genotype groups at Week 53 were not statistically significant. The mean percent changes from baseline in ILS scores at Week 53 were −10.1% for the CD/LR group, 1.6% for the FS/SSM group, and −27.5% for the MS group. The difference was statistically significant for the CD/LR group vs the MS group (*p* = 0.030) and for the FS/SSM group vs the MS group (*p* = 0.013), but was not statistically significant for the CD/LR group vs the FS/SSM group (*p* = 0.64).

#### Spleen volume

**Figure 4:**
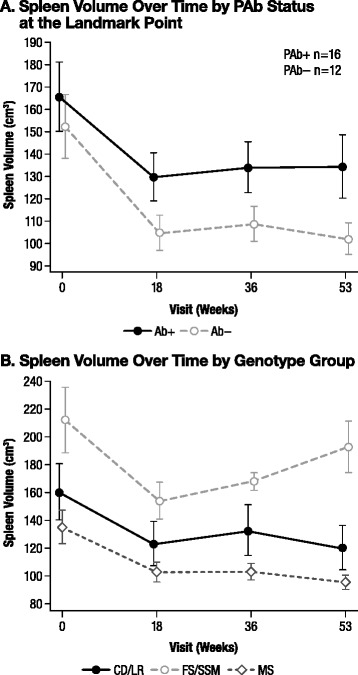
Spleen volume over time by PAb status and genotype group. **A)** Spleen volume over time by PAb status at the landmark point. There are no statistically significant differences at any time point. **B)** Spleen volume over time by genotype group. Week 0, FS/SSM vs MS, *p* = 0.007. Week 18, FS/SSM vs MS, *p* = 0.007. Week 36, FS/SSM vs MS, *p* = 0.002. Week 53, FS/SSM vs MS, *p* = 0.002. Week 53, CD/LR vs FS/SSM, *p* = 0.034. *CD/LR*, complete deletion/large rearrangement; *FS/SSM*, frameshift/splice site mutation; *MS*, missense mutation; *PAb+*, persistently antibody positive; *PAb−*, persistently antibody negative.

We compared the mean spleen volume over time by Ab status at the landmark point. All patient groups regardless of Ab status experienced an initial decrease in mean observed spleen volume on treatment (Figure 4A). In contrast to the results seen for the ILS, the decrease in mean observed spleen volume was maintained to the end of the study, except for in the PNAb+ group, in which there was a slight upswing at Week 53. At the majority of time points, the mean observed spleen volume was slightly higher in the PAb+ group than in the PAb− group, but this difference was not statistically significant (*p* = 0.25 at Week 53). Similar results were seen for the Ab+ vs Ab− (*p* = 0.14), NAb+ vs NAb− (*p* = 0.41), and PNAb+ vs Not PNAb+ (*p* = 0.23) groups at Week 53 (data not shown).

In the genotype analysis, the mean spleen volume at Week 53 was similar between the CD/LR and the MS genotype groups, but larger in the FS/SSM group (Figure 4B). The difference was statistically significant for the CD/LR group vs the FS/SSM group (*p* = 0.034) and for the FS/SSM group vs the MS group (*p* = 0.002), but the difference was not statistically significant for the CD/LR group vs the MS group (*p* = 0.23). The mean percent change from baseline in spleen volume was −25.3% for the CD/LR group, −2.2% for the FS/SSM group, and −23.7% for the MS group. The difference was statistically significant for the CD/LR group vs the FS/SSM group (*p* = 0.040) and for the FS/SSM group vs the MS group (*p* < 0.001), but the difference was not statistically significant for the CD/LR group vs the MS group (*p* = 0.69).

#### Urinary GAG level

**Figure 5:**
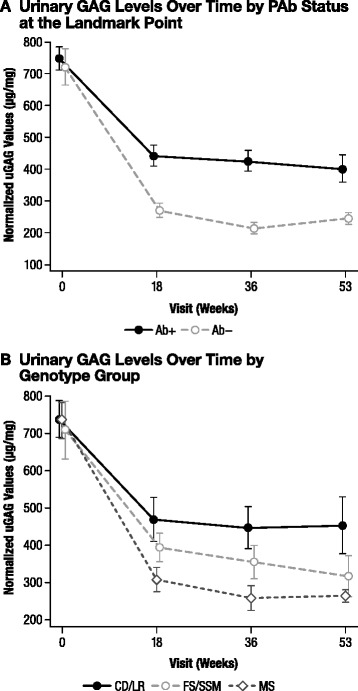
Urinary GAG levels over time by PAb status and genotype group. **A)** Urinary GAG levels over time by PAb status at the landmark point. Weeks 18 and 36, PAb+ vs PAb−, *p* < 0.001. Week 53, PAb+ vs PAb−, *p* = 0.002. **B)** Urinary GAG levels over time by genotype group. Week 18, CD/LR vs MS, *p* = 0.01. Week 36, CD/LR vs MS, *p* = 0.005. Week 53, CD/LR vs MS, *p* = 0.015. There were no other significant differences between groups. *CD/LR*, complete deletion/large rearrangement; *FS/SSM*, frameshift/splice site mutation; *MS*, missense mutation; *PAb+*, persistently antibody positive; *PAb−*, persistently antibody negative*; uGAG*, urinary glycosaminoglycan.

All patients experienced decreased uGAG levels after initiation of idursulfase treatment (Figure 5A). A landmark analysis performed by PAb status at the landmark point showed that patients who became PAb+ had a statistically significantly higher mean uGAG level than did their PAb− counterparts at Week 53 (*p* = 0.002). Similar results were seen for Ab+ vs Ab− (*p* = 0.033), NAb+ vs NAb− (*p* < 0.001), and PNAb+ vs Not PNAb+ patients (*p* < 0.001) at Week 53 (data not shown).

As was done for liver size, percent change from baseline in uGAG concentration at Week 53 was also compared between Ab status groups as adjusted for genotype using analysis of covariance. This was done to determine whether there was any residual relationship between uGAG level and Ab status beyond that explainable by genotype. After adjustment for genotype, only the comparisons involving NAb status groups remained significant at Week 53 (Ab+ vs Ab- adjusted *p* = 0.426; PAb+ vs Not PAb+ adjusted *p* = 0.231; NAb+ vs NAb- adjusted *p* = 0.002; PNAb+ vs Not PNAb+ adjusted *p* = 0.004; data not shown).

The genotype analysis (Figure 5B) showed that the mean uGAG level at Week 53 was significantly higher for the CD/LR group than it was for the MS group (*p* = 0.015). The difference in the mean uGAG level at Week 53 was not significant for the CD/LR group vs the FS/SSM group (*p* = 0.21) or for the FS/SSM group vs MS group (*p* = 0.28). The mean percent change in uGAG level from baseline (adjusted for baseline values) was −40.5% for the CD/LR group, −57.3% for the FS/SSM group, and −62.6% for the MS group. The difference was statistically significant for the CD/LR group vs the MS group (*p* = 0.004), but the difference was not significant for the CD/LR group vs the FS/SSM group (*p* = 0.080) or for the FS/SSM group vs the MS group (*p* = 0.29).

## Discussion

Enzyme replacement therapy in lysosomal storage diseases leads to production of Abs in fractions of the populations that range from about 2–15% (Gaucher disease), to 95–100% (MPS IV and Pompe disease) [10,23]. Purely descriptive analyses of Ab formation in patient groups cannot answer the question of whether the presence of Abs has clinical significance, and if so, what type of strategies should be implemented to mitigate this impact. In Pompe disease, for instance, a subgroup of patients (CRIM-negative) develop high-titer NAbs, which are clearly associated with worse clinical outcomes [9]. In this particular population, immunomodulatory regimens are now becoming standard of care, in order to prevent the production of Abs and ensure a good therapeutic response [24]. In MPS IV, initiation of ERT leads to a universal Ab response, but there appears to be no correlation between Ab titer and clinical outcomes [10]. In Fabry disease, there appears to be no correlation between titers of anti-drug Abs and the onset of clinical events, glomerular filtration rate, or plasma biomarkers [25].

For MPS II, we conducted an earlier analysis [11] that showed no association between Ab status and safety or efficacy outcomes. That study; however, had only enrolled patients with the attenuated form of the disease, and consequently there were no patients with the CD/LR genotype included in the analysis. We have now expanded our investigation to include a population of pediatric MPS II patients with the severe phenotype and CD/LR genotype. In HGT-ELA-038, 28 treatment-naïve MPS II patients who were ≤5 years of age at screening received 0.5 mg/kg of idursulfase weekly for 52 weeks [14]. In our current post hoc analysis of the HGT-ELA-038 data, we have rigorously examined the relationship between anti-idursulfase Ab status, genotype, and safety and efficacy outcomes by distinguishing between events happening prior to and after seroconversion.

We have demonstrated that there is an association between genotype and Ab response in this cohort. Patients with the CD/LR genotype were more likely to develop an immune response to idursulfase than were the patients with the MS genotype (Table 3). Patients with the FS/SSM genotype fell between the other 2 genotype groups. These results make biological sense: patients who produce no enzyme at all are more likely to develop an immune response following idursulfase treatment than are those patients with a milder mutation that produces a truncated protein or a protein with a single amino acid substitution. With no endogenous enzyme, idursulfase would be viewed as a foreign, non-self protein by the body.

The safety data were analyzed both by Ab status and by genotype. Antibody status was not associated with time to a first TEAE or SAE after the landmark point, nor was Ab status associated with any statistically significant overall increase in TEAE or SAE risk. For SAEs, several statistical methods indicated that the risk of SAEs was higher in patients who developed Abs, but the differences were not statistically significant. It is interesting to note that the majority of SAEs in this study could be considered disease-related, such as respiratory infections. Only 10 of the 38 SAEs (26%) were considered at least possibly related to study drug. All 10 SAEs were also IRAEs and were reported by only 4 patients, all of whom were in the CD/LR genotype group (Table 2). Therefore, the numerically higher cumulative probability of developing an SAE in the CD/LR group compared with the MS group may reflect the more serious disease pathology in the former group. Because the patients with the CD/LR genotype invariably developed Abs (Table 3), any apparent increase in risk for SAEs associated with Abs could be explained by the more severe underlying disease phenotype that is driven by genotype.

There did not seem to be an association between Ab+ status and risk of IRAEs. For IRAEs, landmark analyses among patients who were IRAE-naïve at the landmark point found that patients with any type of positive Ab status were no more likely to experience future IRAEs than were patients who did not have that positive Ab status (Figure 2). The event rate for IRAEs was higher for patients who became Ab+ than it was for those who remained negative throughout the study. Of interest, the rate was highest during the Ab− period, and then decreased over time, even as the patients became Ab+ or NAb+, or PAb+ or PNAb+. A similar phenomenon was seen in our previous post hoc analysis of older patients with the attenuated form of MPS II [11]. This effect is likely due to the implementation of preventive measures [13] after a first IRAE rather than any protective effect of Abs. This pattern suggested that certain genotypes, an intrinsic factor, may predispose patients to both Ab formation and to IRAEs, rather than Abs being the cause of IRAEs. Indeed, at the end of the study, the cumulative probability of having at least one IRAE was highest for patients in the CD/LR group (87.5%) and lowest (46.2%) for patients in the MS group, with a shorter time to development of a first IRAE for CD/LR patients than for MS patients (*p* = 0.004). Notably, all 4 patients who reported IRAEs that qualified as SAEs were in the CD/LR group.

Efficacy variables were also analyzed by Ab status and genotype. All patient groups experienced an initial decrease in liver size after initiation of idursulfase. Overall, Ab+, PAb+, NAb+, and PNAb+ patients demonstrated an upswing in liver size toward the end of the study (Study Week 53), returning to approximately baseline values (Figure 3A), and the differences were significant. To determine whether there was any residual relationship between liver size outcome and Ab status beyond that explainable by genotype, the percent change from baseline in ILS at Week 53 was also compared between Ab status groups as adjusted for genotype using analysis of covariance. After adjustment for genotype, only the comparisons between NAb status groups remained significant. In the genotype-based analysis, patients in the CD/LR and FS/SSM groups had a less pronounced decrease in liver size than did those in the MS group (Figure 3B). The CD/LR and FS/SSM groups also tended to return towards baseline values at the end of the study. For spleen size, all patient groups also experienced a decrease in spleen size after initiation of idursulfase. Unlike for liver size, Ab status had no statistically significant association with the spleen size response (Figure 4A). In the genotype analysis, the mean spleen volume at Week 53 was similar between the CD/LR and the MS genotype groups, but significantly greater in the FS/SSM group than in either of the other groups (Figure 4B). These results suggest that Ab status and genotype may influence the degree of liver size response to idursulfase and that genotype may influence the degree of spleen size response to idursulfase.

Urinary GAG levels fell after the initiation of idursulfase for all patient groups, but patients with Ab+, PAb+, NAb+, and PNAb+ status had a less robust uGAG decline in the latter part of the study than did their Ab− counterparts (Figure 5A). As with liver size; however, comparisons between Ab status groups for the percent change from baseline in uGAG level at Week 53 only remained significant for NAb status groups after adjustment for genotype. The comparisons between the Ab+ and Ab− groups and PAb+ and Not PAb− groups were no longer significant. In the genotype-based analysis, patients in the CD/LR genotype group had a less pronounced decrease in uGAG levels than did patients in the MS group (Figure 5B). Overall, the efficacy of idursulfase as measured by significantly decreased uGAG levels from baseline was maintained over the course of the study. The continued association of NAbs with poorer outcome measures suggest that NAbs, as expected, are effective at inactivating I2S, thereby reducing the effect of idursulfase therapy.

There are a number of limitations to our data analyses that are inherent to the study design. First, although this is one of the largest cohorts of MPS II patients that has been studied for the association between genotype, Ab status, and safety outcomes, the numbers are still small. Conclusions should be considered with this fact in mind. Second, the exact time of each patient’s seroconversion was not known. Therefore, we assigned seroconversion dates according to the algorithm described in the Methods. The choice of the assignment date of Calculated Week 4.5 was made based on the fact that it takes several weeks for an Ab response to develop after exposure to a new antigen [19]. Calculated Week 4.5 was thus halfway between first exposure (baseline) and Study Week 9, at which time point all patients who would become Ab+ in this study had done so. In some cases, data indicating that a patient had seroconverted prior to Calculated Week 4.5 were available because of an unscheduled sampling taken after an IRAE. In those cases, the earlier time point—the date of the unscheduled sampling—was used as the seroconversion date. Third, MPS II is a complex multi-systemic disease; therefore, many of the observed SAEs may be related to the underlying disease and not necessarily to a drug effect, which further complicates the interpretation of drug safety data in this population.

## Conclusions

We have rigorously examined the relationship between anti-idursulfase Ab status, genotype, and safety and efficacy outcomes for MPS II patients aged ≤5 years at screening who were treated with idursulfase as part of study HGT-ELA-038. Our data support the idea that patient genotype is associated with the risk of an immune response to idursulfase. Patients in the CD/LR group were the most likely to develop Abs, while patients in the MS group were the least likely to develop Abs. Our study also provides evidence that patient genotype, but not Ab status, is associated with the overall safety of idursulfase treatment. A CD/LR genotype appeared to be associated with an increased risk of IRAEs and SAEs when compared with the MS genotype. Therefore, any apparent associations between Ab status and safety outcomes are most likely influenced by genotype. Both NAbs and genotype seem to be associated with a less robust efficacy response to idursulfase treatment at Week 53 in terms of liver size and uGAG level. In contrast, genotype alone appeared to be associated with spleen size outcome on idursulfase treatment.

We would like to emphasize that the associations between Ab formation, genotype, and efficacy outcomes were not universally observed. For example, individual patients with a CD/LR genotype and high Ab titers could still experience a large decrease in liver size, spleen size, or uGAG level. Because of this observation, genotype and Ab status appear to have limited value in predicting a patient’s clinical response and must be interpreted with caution. As in our previous post hoc analysis of data from the phase II/III trial and extension study of idursulfase in attenuated MPS II patients over 5 years of age [11], our data do not suggest specific recommendations or modifications be made to the current treatment guidelines associated with the use of idursulfase.

## Abbreviations

AAL: Anterior axillary line
Ab+: Antibody positive
Ab−: Antibody negative
AEs: Adverse events
CD/LR: Complete deletion/large rearrangement
CSA: Conformation-specific antibody
ELISA: Enzyme-linked immunosorbent assay
FS/SSM: Frameshift/splice site mutation
GAGs: Glycosaminoglycans
I2S: Iduronate-2-sulfatase
IgG: Immunoglobulin G
ILS: Index of liver size
IRAEs: Infusion-related adverse events
MPS: Mucopolysaccharidosis
MS: Missense mutation
NAb+: Neutralizing antibody positive
NAb−: Neutralizing antibody negative
Not PAb+: Not persistently antibody positive
Not PNAb+: Not persistently neutralizing antibody positive
PAb+: Persistently antibody positive
PNAb+: Persistently neutralizing antibody positive
RIP: Radioimmunoprecipitation
SAEs: Serious adverse events
STL: Sternal line
TEAEs: Treatment-emergent adverse events
uGAG: Urinary glycosaminoglycan
